# Enabling Angioplasty‐Ready “Smart” Stents to Detect In‐Stent Restenosis and Occlusion

**DOI:** 10.1002/advs.201700560

**Published:** 2018-02-16

**Authors:** Xing Chen, Babak Assadsangabi, York Hsiang, Kenichi Takahata

**Affiliations:** ^1^ Department of Electrical and Computer Engineering University of British Columbia Vancouver BC V6T1Z4 Canada; ^2^ Department of Anesthesiology and Critical Care Medicine The Johns Hopkins School of Medicine Baltimore MD 21287 USA; ^3^ Department of Surgery Vancouver General Hospital University of British Columbia Vancouver BC V5Z1K3 Canada

**Keywords:** angioplasty, restenosis, smart medical implants, stents, wireless sensing

## Abstract

Despite the multitude of stents implanted annually worldwide, the most common complication called in‐stent restenosis still poses a significant risk to patients. Here, a “smart” stent equipped with microscale sensors and wireless interface is developed to enable continuous monitoring of restenosis through the implanted stent. This electrically active stent functions as a radiofrequency wireless pressure transducer to track local hemodynamic changes upon a renarrowing condition. The smart stent is devised and constructed to fulfill both engineering and clinical requirements while proving its compatibility with the standard angioplasty procedure. Prototypes pass testing through assembly on balloon catheters withstanding crimping forces of >100 N and balloon expansion pressure up to 16 atm, and show wireless sensing with a resolution of 12.4 mmHg. In a swine model, this device demonstrates wireless detection of blood clot formation, as well as real‐time tracking of local blood pressure change over a range of 108 mmHg that well covers the range involved in human. The demonstrated results are expected to greatly advance smart stent technology toward its clinical practice.

## 1 Introduction

Cardiovascular disease (CVD) is the number one leading cause of mortality worldwide. For example, in the United States, more than two thousand patients die from CVD each day.^1^ The main pathology of CVD is atherosclerosis, the hardening of blood vessels caused by plaque buildup on the arterial wall, which progressively narrows arteries to obstruct blood flow and thus interrupt normal oxygen and nutrient supply.^2^ If severe, this leads to heart attack and stroke. One of the most common treatments is stenting. Stents are metallic tubular implants that are permanently placed in narrowed arteries to physically prop open and scaffold the vessels to restore blood flow.^3^ Backed by its clinical efficacy, millions of stents are implanted every year. The presence of a metallic stent within an artery, however, can cause inflammation, leading to excess growth of arterial tissue that may cause renarrowing within the stent. This complication is known as in‐stent restenosis.^4^ The likelihood of in‐stent restenosis can reach as high as 50% among stented patients.^5^ Drug‐eluting stents, or stents coated with medications to be released slowly to suppress cellular proliferation, are currently used to prevent restenosis at an early stage after stenting. Although beneficial within the first few years post implantation, covered stents may pose an increased risk of late thrombosis and resultant heart attacks over time.^6^ The two primary methods used to identify in‐stent restenosis in arteries, duplex ultrasound and angiography,^7^, ^8^ are usually not done unless patients present with chest pain or other symptoms.^9^

The need for a rapid, noninvasive, and easily accessible method to detect in‐stent restenosis has led to the development of “smart” stents with self‐sensing and communication ability via integration of micro‐electromechanical systems (MEMS), microelectronics, and antenna functions with stents.^10^, ^11^, ^12^, ^13^, ^14^ This technology targets remote monitoring of implanted stents that can wirelessly transmit sensed signals in real time. The smart stent system can be configured to receive a signal detected by the implant upon restenosis or thrombosis using a portable reader to alert the need for detailed diagnosis and possible treatment. Both passive sensing stent, based on different sensing principles (e.g., radiofrequency (RF) inductor–capacitor (*LC*) resonant tank coupled with MEMS capacitive pressure sensors, and magnetoelastic resonator), and active sensing stent integrated with application‐specific integrated circuit and sensor chips have been proposed and widely studied.^10^, ^15^, ^16^ Unfortunately, no related attempts have reached successful in vivo demonstration of their target functions. This outcome may be associated with the following factors. First, the proposed designs only considered engineering aspects without addressing their utility in clinical settings, such as compatibility with standard angioplasty and stenting procedures (known as percutaneous coronary intervention (PCI)).^17^ For example, although the first smart stent achieved the inductive antenna function through its design based on micropatterned alloy foil,^10^ this type of stent, with a planar‐like shape at the initial (pre‐expansion) state, cannot be inserted and guided into a blood vessel through a standard stenting procedure. Second, it is impractical to place the IC and sensor chips on the outer surface of stent,^18^ due to (i) the destruction of chips during stent crimping, a harsh mechanical step for catheter assembly that involves radial compression forces of up to 1000 N (e.g., J‐Crimp, Blockwise Engineering LLC, USA), and (ii) the presence of objects on the stent's outer surface is clinically unacceptable as it does not allow insertion and navigation of the device into/through blood vessels without injuring the blood vessel. Third, chip packaging and integration on stents using adhesives or similar methods^19^ are not reliable. In one attempt of animal testing of a smart stent,^20^ signaling failure occurred after implantation due to breakage of both sensor and its bonding seam. In another work on a sensor‐integrated telemetric stent device,^21^ the wireless sensing function of the device was lost during its animal model testing, which was later identified by a failure of the conductive adhesive joint between the sensor and the stent when mechanical distortions were applied on the device during operation. It is evident that for clinical practice, a more robust sensor and careful packaging are required to survive the harsh mechanical steps (i.e., stent crimping, insertion, and expansion) and to function over a long period in vivo.^22^ In addition, for the sensor‐integrated stent based on passive resonator,^10^ the relatively low quality (Q) factor remains as an issue in providing wirelessly detectable signals to an external receiver.

Here, we advance smart stent technology by achieving its compatibility with the standard PCI procedure through enhancing the electromechanical performance of the sensor‐integrated stent device, and demonstrating its expected functionality using a swine model. The primary focus of this work with respect to its engineering aspect is to build a balloon‐expanding smart stent integrated with a MEMS sensor that simultaneously offers both high‐performance wireless sensing function and mechanical robustness. To this end, we develop a stainless‐steel chip of capacitive pressure sensor and its microwelding‐based packaging on stents.^23^ From a physiological and clinical perspective, the device is constructed and encapsulated with biocompatible materials, while being designed to comply with standard catheterization procedures enabled with high mechanical robustness and compact footprint of the developed device. Since stenting is a personalized treatment, stent selection depends on the diameter of the particular artery and the length of diseased vessel needing to be treated. As stents with different dimensions have been reported to exhibit different restenosis rates,^24^ we also evaluate the sensing characteristics of the smart stent devices with different diameters and lengths as part of the prototype development.

## 2 Results and Discussion

### 2.1 Working Principle of Sensor‐Integrated Wireless Stent System

**Figure 1:**
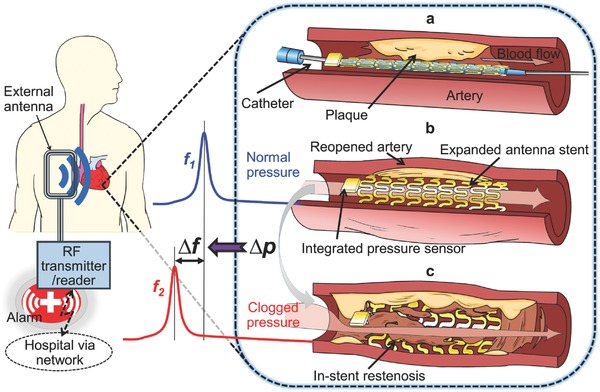
Conceptual schematic of the smart stent and its functions. a) A pressure‐microsensor‐integrated wireless stent crimped on the balloon catheter is positioned at the targeted stenosis site in the artery. b) The smart stent is deployed by balloon inflation to start self‐diagnosing while scaffolding the narrowed artery after removal of the catheter; the stent's resonant frequency is at its nominal level (*f*_1_). c) In‐stent restenosis changes local blood pressure and shifts the stent's frequency (to *f*_2_) as a sign of the problem; the implant is continuously monitored through a handheld wireless reader that sends out a warning of restenosis upon occurrence.

**Figure** **1** illustrates a representative scenario of how the smart stent, assembled on a balloon catheter, is implanted to treat an artery narrowed by plaque deposition and to detect early signs of in‐stent restenosis as it develops. Following the PCI procedure, a pressure‐sensor‐integrated stent is firmly crimped onto the balloon catheter (Figure 1a) and guided to the site of stenosis through a sheath (a valved vascular tube used to secure a passageway to an artery while preventing blood from flowing out); the balloon is then inflated to expand and deploy the stent that pushes the plaque against the artery wall, widening the narrowed blood vessel and restoring normal blood flow. Deflating and retracting the balloon catheter leaves the stent implanted permanently to scaffold the artery together with the integrated sensor (Figure 1b). The stent in this system is designed to function not only as a typical mechanical scaffold but also as an electrical inductor or antenna. Using a capacitive type of MEMS pressure sensor that works as a variable capacitor over pressure, a combination of the stent and the sensor integrated at one end of the stent forms a *LC* tank, whose resonant frequency (inversely proportional to *C* ^1/2^) represents the on‐site condition of surrounding blood pressure, which may be interrogated using a handheld wireless reader with an external antenna by establishing inductive coupling with the antenna stent.^25^ Upon the onset of in‐stent restenosis when excess tissue or plaque builds up on the inner walls of the stent to obstruct blood flow, hemodynamics and local pressure distribution around the stent change (Figure 1c), and accordingly, the resonant frequency shifts away from its baseline as an indication of in‐stent restenosis. This change provides critical information that guides the patient to further examination and treatments at an earlier stage. The passive device architecture removes the need for battery and active circuitry, allowing the entire device to be compact enough to fit into the small artery (e.g., 3–4 mm in diameter for coronary artery).

### 2.2 Design and Fabrication of Smart Stent

**Figure 2:**
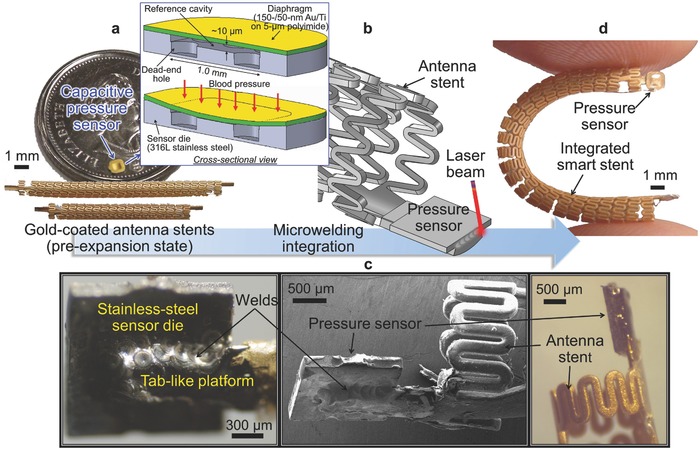
Fabrication process and fabricated prototype. a) Optical image of fabricated MEMS capacitive pressure sensor chip (on a Canadian quarter, with an inset showing the sensor's design and structure) and two gold‐covered inductive stents with 30 mm length (upper) and 20 mm length (lower). b) Schematic of laser microwelding process joining the sensor chip onto the stent. c) Close‐up photographs (left and right) and scanning electron microscope image (middle) showing microwelded joint between the chip and the stent. d) Finalized smart stent device (showing 30 mm version).

**Figure** **2** displays the construction and integration process that we developed for the advanced smart stent. The stent device is comprised of two main components, an inductive stent and a microcapacitive pressure sensor, as shown in Figure 2a. The inductive stent, custom‐designed to have an overall helical structure of medical‐grade stainless steel (type 316L, the most commonly used stent material),^26^ provides orders‐of‐magnitude higher electrical inductance to serve as a RF antenna, while offering higher radial stiffness and axial compliance compared with commercial stents.^27^ This work adopts and tests the antenna stents with two different axial sizes, 20 and 30 mm, to investigate their sensing characteristics. These stents are electroplated with gold to decrease the series resistance of the stent and thus enhance its Q factor,^27^ and then insulated by conformal coating of Parylene C, an FDA‐approved biocompatible material,^28^ with a relatively large thickness (20 µm) to preserve the antenna functionality by suppressing signal damping while residing in conductive blood environment.^21^ It is worth noting that both Parylene C and gold have been used in commercial stents (respectively as a drug‐eluting base coating material and a radiopaque layer for the implants).^26^ The MEMS capacitive pressure sensor used for the smart stent construction is also custom‐designed and microfabricated (Figure 2a). This gauge pressure sensor is comprised of a microchip of the same stainless‐steel as the antenna stent, which serves as the sensor's substrate as well as the fixed capacitive electrode, and a gold–titanium‐polyimide multilayered diaphragm serving as the other electrode that is deformable. The sensor chip has a small footprint (1.5 × 1.5 mm^2^) and low profile (200 µm thick) to minimize the disturbance of normal blood flow, and is made of all biocompatible materials.

For sensor integration with the antenna stent, we use laser microwelding for these two stainless‐steel parts (Figure 2b) instead of conductive epoxy as reported previously,^10^, ^21^ to exploit both engineering and physiologic benefits that the technique offers. On the engineering side, laser microwelding yields higher mechanical robustness at the joint between the sensor and the stent compared with those made by adhesives. The microwelding process also offers lower electrical resistance at the joint as it establishes direct metal‐to‐metal joints while removing the need for intermediate layers such as conductive adhesive, contributing to increasing the Q factor of the *LC* tank, i.e., the sensor‐stent combination. For example, microwelded bonds showed 2× larger shear strength and 6× higher electrical conductance when compared with those of conductive epoxy as we reported.^23^ In addition, the welding approach potentially improves fabrication efficiency, as it forms a bond instantly upon laser beam shooting, while epoxy adhesives typically require tens of minutes or more to be cured. On the physiological side, microwelds offer high resistance against corrosion, especially galvanic corrosion known to be one of the main failure mechanisms of implant devices,^29^ and demonstrated a long‐term reliability when exposed to inner human body fluids when compared to epoxy.^30^ Figure 2d shows an example of completed 30 mm long device. As evident from the comparison between Figure 2c and Figure S1 (Supporting Information), this alternative packaging method enables fusion of micro regions of the sensor die and the stent to create a continuous, low‐profile, and robust bond. The resultant device potentially minimizes blood flow disturbance, and mechanical failure during a PCI procedure as will be discussed in the next section.

### 2.3 Bench Testing with Standard Angioplasty Procedure

**Figure 3:**
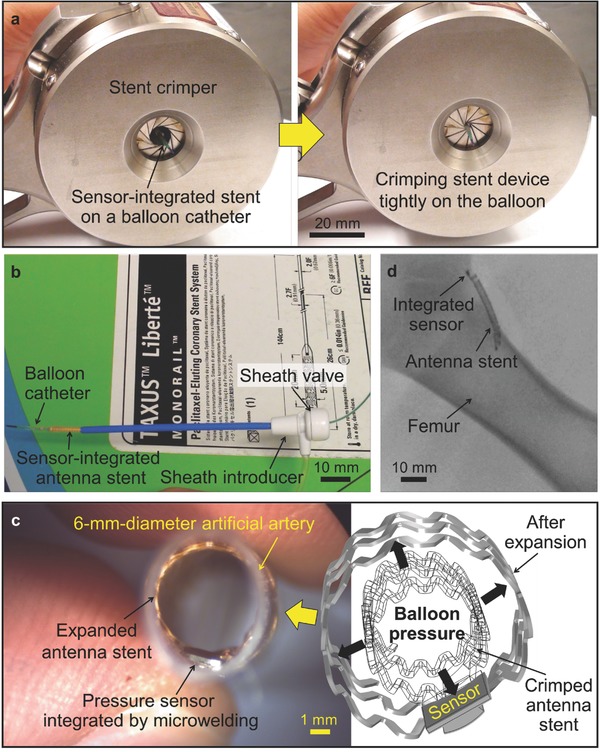
Bench testing of smart stent. a) Mechanical crimping on the balloon catheter. b) Guiding the combined smart stent and balloon catheter through a sheath. c) Deployment of the stent into an artificial artery by inflating the balloon catheter. d) X‐ray imaging of the radiopaque stent device.

In order to translate this technology from bench to bedside, the smart stent device needs to meet the same standard as commercial stents. In light of this, we assessed the compatibility of fabricated prototypes with a PCI procedure and relevant devices through bench testing. For delivery testing, two types of commercial balloon catheter systems with different specifications (Low Profile PTA Balloon Dilatation Catheter, Cook Medical Inc., IN, USA, and Paclitaxel‐Eluting Coronary Stent System, Boston Scientific Co., USA) were used depending on the stent's length and diameter. Initially, a prototype smart stent was mounted onto the balloon region of the catheter (see Figure S2, Supporting Information). This was followed by the use of a stent crimper (HH100 SS, Machine Solution Inc., USA) to exert a radial compression force to the stent device (**Figure** **3**a) to reduce its initial diameter and crimp it onto the balloon. Radial forces in the order of 100 N were applied to achieve tight crimping. This is a critically important factor for proper and successful device delivery, as with insufficient crimping, the stent could fully or partially slip off from the balloon catheter when passed through the sheath or blood vessel, leading to failure of stent delivery.

After crimping, the smart stent (now mounted on the balloon catheter) has to be inserted through a sheath in order to reach the artery. This requires forcibly passing through a hemostatic valve. As shown in Figure 3b, 20 and 30 mm long versions of the smart stent, crimped on balloon catheters, were verified to easily pass through a commercial sheath introducer (Check‐Flo Performer Extra Large Introducers, Cook Medical Inc., IN, USA; 2 mm inner diameter) with no apparent interference to the device both mechanically and electrically. This was achieved through two main advantages of the adopted microwelding approach for sensor integration, i.e., high mechanical robustness of the bond against shear stress applied when passing through the hemostasis valve, and the small footprint and low profile of the resultant device that enabled smooth insertion with minimized guiding resistance.

Passing through the sheath‐valve channel, the device on the balloon catheter was delivered into a silicone‐based artificial artery (Dynatek Labs, MO, USA) for the final deployment. Deionized water was injected into the balloon with an insufflator (Cook Sphere, Cook Medical Inc., IN, USA) to fully inflate the balloon with hydraulic pressure up to 16 atm. This full inflation was reached in three steps, so that the stent device was expanded to three different diameters, i.e., 4, 5, and 6 mm. Figure 3c shows a 30 mm length device that was expanded in a 6 mm diameter artificial artery with a wall thickness of 0.2 mm. X‐ray imaging of an integrated device deployed and implanted inside a pig model (Figure 3d, performed in one of in vivo tests discussed later) demonstrates high radiopacity of the device brought by both the gold coating on the stent surfaces and the gold layer of the sensor diaphragm.

The laser‐microwelded prototypes (either 20 mm length or 30 mm length version; 16 samples tested) showed ≈80% success with no evidence of mechanical or electrical failure, while those integrated with conductive epoxy (CW2400, Chemtronics, GA, USA) showed frequent breakaway of the sensors from the stents when experiencing the same steps. We also verified that the microwelded samples also provided notably higher long‐term reliability than conductive epoxy joint through accelerated aging test (see the Supporting Information regarding the comparative experiment and results). These results indicate that this new generation of smart stent is highly robust and potentially useful for clinical practice.

### 2.4 In Vitro Electrical and Wireless Characterization

**Figure 4:**
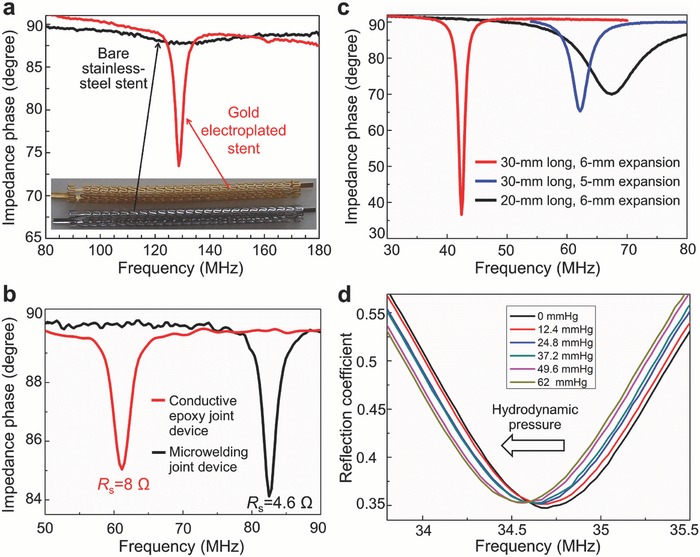
Measurement results from in vitro testing. a) Impedance phase dip comparison between bare and gold‐electroplated inductive stent connected with a 10 pF discrete capacitor. b) Impedance phase dips and serial resistances of two 30 mm long stents (4 mm in expanded diameter) joined with capacitive pressure microsensors by conductive epoxy and laser microwelding. c) Impedance phase dips of three differently sized microwelded smart stents in vascular grafts. d) Resonant frequency shifts of a 30 mm long microwelded smart stent (6 mm in expanded diameter) over varying applied pressure in water flow.

One essential path to enhancing wireless sensing resolution is to raise the Q factor of the resonant tank,^25^ which can be effectively addressed by decreasing its parasitic resistance. For the present device, parasitic resistance mainly originates in the inductive stent and the joint between the sensor and the stent. As discussed in the preceding section, these two major components of resistance were reduced by gold coating on the stent structure using electroplating and by microwelding the sensor to the stent, respectively. Their impacts on the Q factor were evaluated. To assess the effect of gold coating, two 30 mm long, unexpanded inductive stents, one with and the other without the gold layer (see Figure S3, Supporting Information), were coupled with the same 10 pF capacitor to form *LC* circuits, and their frequency spectra of inductive phase were wirelessly measured using a spectrum‐impedance analyzer (4396B, Agilent Technologies, CA, USA) via inductive coupling as shown in Figure S4 (Supporting Information). Both samples exhibited almost identical resonant frequencies (≈130 MHz) while significantly different amounts of the phase dip or Q factor (**Figure** **4**a). The gold‐plated case clearly showed a notably stronger resonance, with a Q factor of 43 that was >10× higher than that of the bare inductive stent. This outcome agrees with the relationship of their measured parasitic resistances, verifying the importance of relatively thick gold (or highly conductive metal) coating on the stainless‐steel stent to exploit the skin effect (as discussed in the Experimental Section).

To evaluate the effects of microwelding on the resonance, another set of two 30 mm long inductive stents with gold‐plated layers (whose parasitic resistances were approximately identical) were coupled with fabricated capacitive pressure sensors (with different base capacitances associated with fabrication variation), one with laser microwelding and the other with the conductive epoxy for comparison. The two ends of the open device (i.e., series *LC* circuit) were connected with the impedance analyzer to read its serial resistances (*R*_S_) for both devices at their resonant frequencies; after closing the circuit, they were expanded to 4 mm in diameter using a balloon catheter to characterize their resonances in the wireless manner same as the above measurement. The results (Figure 4b) suggested that the microwelded device provided nearly 2× higher conductance or Q factor than those of the epoxy‐bonded device (4.6 vs 8 Ω, and 39 vs 21, respectively), apparently owing to the electrical merit offered by the microwelding method. These two tests proved that the combination of gold coating and laser microwelding used for the device fabrication and integration was highly effective in raising the Q factor of the device.

Understanding the wireless characteristics of this type of smart stent when used for different sized arteries is of importance for the technology to be clinically viable. In light of this, frequency responses of the devices were evaluated using graft‐deployed samples of different lengths and diameters, i.e., a 30 mm long device fully expanded in 6 mm diameter vascular grafts (GORE‐TEX Stretch Vascular Graft, W. L. Gore & Associates, Inc., AZ, USA), a 20 mm long device also fully expanded in the same graft, and another 30 mm long device expanded in a 5 mm diameter graft of the same type. Figure 4c shows the impedance phase dips produced by these three samples measured with an identical external coil arrangement in air. The 30 mm sample in the 6 mm diameter graft shows the largest dip amplitude (of 53°) in resonance at the lowest frequency (of 42 MHz), followed by the 30 mm sample in the 5 mm diameter graft (25° dip) at a mid‐level of resonant frequency (62 MHz), and the 20 mm sample in the 6 mm diameter graft (20° dip) at the highest resonant frequency (67 MHz). These variations and trends of resonant amplitude and frequency are consistent with the stents' inductances of the samples (and resultant coupling coefficients) that depended on their lengths or numbers of turns as well as on the cross‐sectional areas of the stents (as they essentially behave as solenoid coils).^27^ This result clearly suggests that the use of longer and wider stents for the proposed smart stent construction is beneficial in terms of raising its wireless sensing ability. Clinically, stent size is dependent on the anatomy of the underlying vessel and pathology. As in‐stent restenosis depends on the stent's dimensions,^24^ this affects the size selection as well. Therefore, to achieve stable wireless reading of implanted smart stents of varying sizes, the reader system will need to calibrate sensed signals and compensate for their variations caused by the device sizes.

Wireless hydrodynamic pressure sensing was tested on the fabricated prototypes in a flow circulation loop (prior to this test, we verified their sensing functionality with hydrostatic pressure using a set‐up shown in Figure S5a, Supporting Information) as illustrated in Figure S5b (Supporting Information). A commercial pump (Haake F423, Thermo Fisher Scientific Inc., MA, USA) was used to force the water to flow through a stent device deployed in the 6 mm diameter commercial graft noted above. The pressure exerting on the device was controllable through two manual valves located upstream and downstream of the device. A reference pressure sensor (24PC, Honeywell Sensing and Control, MN, USA) was connected to the flow loop to read intraluminal pressure applied to the device during the test (Figure S5c, Supporting Information). Frequency response of the device within the graft was wirelessly monitored through an external antenna by tracking a reflection coefficient (*Γ*) magnitude using the spectrum analyzer to which the antenna was connected (through a Bayonet Neill–Concelman (BNC) coaxial cable, to extend the working distance between the analyzer and the antenna). Figure 4d displays the measured response of a 30 mm long, microwelding‐integrated prototype to varying intraluminal pressures produced by water flow (up to 0.75 L min^−1^). The shifts of the resonant frequency were clearly resolved toward lower values (due to increase of the sensor's capacitance) as pressure was increased with an increment of 12.4 mmHg. A total frequency shift of 200 KHz was observed over the full pressure change of 62 mmHg, suggesting an average sensitivity of 93 ppm mmHg^−1^. The pressure increment of 12.4 mmHg was found to represent a pressure resolution that the device could provide with the wireless interface used. It should be noted that this resolution is 5× higher than that of the previously reported stent device tested in air,^21^ even though the present case was measured in the water environment that damped RF signals more than the air ambient case. The threshold reduction of local blood pressure that clinically indicates in‐stent restenosis has been reported to be ≈23 mmHg (in the mean arterial pressure (MAP), calculated from the reported threshold value (0.75) of fractional flow reserve,^31^ a technique to measure pressure drop across arterial stenosis, with a nominal MAP of 93 mmHg). This suggests that with the current pressure resolution, the developed device is expected to be able to detect an early state of restenosis.

### 2.5 In Vivo Experiments

**Figure 5:**
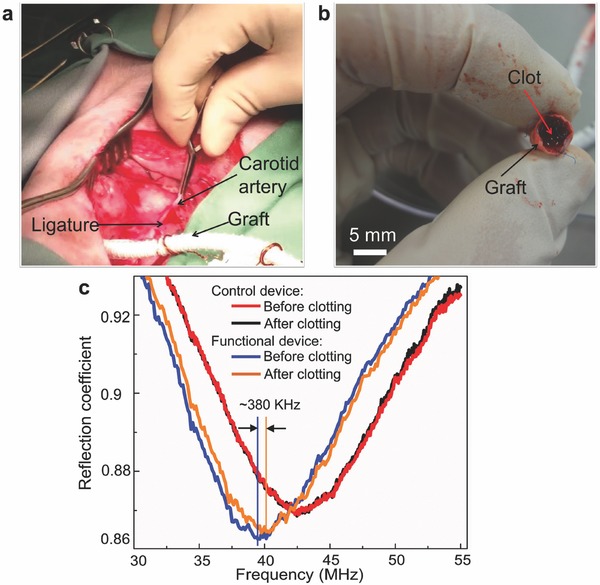
Wireless detection of in‐stent occlusion. a) Bypass surgery of the graft with deployed sensor‐integrated smart stents in the carotid artery of a swine model. b) Blood clot occurred inside the graft during test. c) Measured frequency response of functional and control stent devices to blood clot formation.

The developed prototypes were tested using a swine model under ethics protocol #A11‐0067 approved by the University of British Columbia. A 6 month old, 28 kg female pig was used in this test. During surgery, the pig was fully anesthetized and its vital signs (e.g., electrocardiogram waveform, blood pressure, heart rate, blood oxygen saturation, and inspired carbon dioxide concentration) were constantly monitored using a multiparameter physiologic monitor system (LifeWindow LW6000, Digicare Biomedical Technology Inc., USA). The blood pressure (systolic/diastolic) and heart rate before surgery were measured to be 99/61 mmHg and 115 per minute, respectively. Given the small size of animal's blood vessels, a surgical model was created using GORE‐TEX vascular grafts. In the first test, a 5 mm diameter graft that contained two fully expanded 30 mm long devices, one with functional pressure response and the other with no pressure response serving as a control, was placed in a bypass model (Figure S6a, Supporting Information). For this procedure, following administration of heparin, the carotid artery was ligated in its mid‐section, and the two ends of the graft anastomosed upstream and downstream of the ligature to establish a bypass. Following completion of the bypass, flow was restored through the bypass loop and two external antennae (one as a backup) were looped around the graft (**Figure** **5**a). The antenna was then connected to the spectrum analyzer via a coaxial cable (as used in the in vitro flow test). The RF source power provided from the analyzer was set at 20 dBm. The presurgery resonant frequencies of the functional and control devices at zero gauge pressure were respectively measured to be 62 and 68.5 MHz in air and 40 and 45 MHz in saline (0.15 m phosphate buffered), which shifted to 39 and 42.5 MHz with blood flow (at a blood pressure of 79/52 mmHg and a MAP of 61 mmHg).

As a preliminary test of device's sensing function, blood flow through the graft was mechanically restricted by clamping it while wirelessly monitoring the resonant frequency of the device. When downstream flow was restricted, intraluminal pressure experienced by the functional device lowered its resonant frequency as expected, whereas no frequency shift was observed with the control device. However, after repetitive stoppage of blood flow several times, the functional device became to show no frequency response to the flow restriction. It was visually verified (after incising the graft) that the graft formed a blood clot that blocked the blood flow across the device (Figure 5b). Although this situation did not allow further real‐time sensing, it in fact created a scenario similar to in‐stent restenosis and subsequent thrombosis (clotting of graft). The comparison of the reflection dip spectra observed in the external antenna before and after clot formation (Figure 5c) showed no frequency shift with the control device, whereas the functional device exhibited a frequency shift (≈+380 KHz, obtained by processing the raw spectrum data using MATLAB R2014b, MathWorks Inc., USA) upon clotting, due to a decrease of the sensor's capacitance led by absence of blood flow and pressure in the graft. This preliminary test proved two important functions of the device—its ability to produce pressure‐induced signals that were wirelessly detectable upon occurrence of an obstruction formed in in vivo blood flow, and the robustness of the microwelding‐integrated device in the face of mechanical stress, both of which were not achieved in the previous work.^16^, ^20^, ^21^

**Figure 6:**
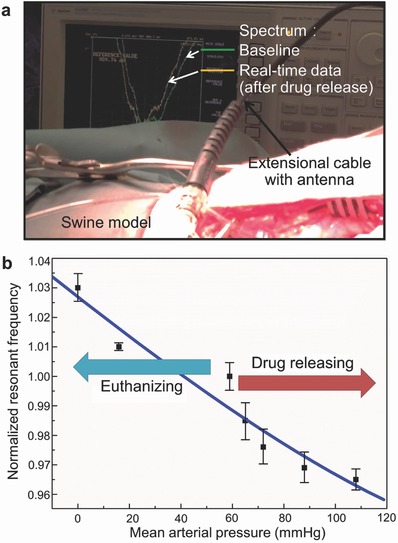
Wireless measurement results from the swine model test. a) Reflection spectra (on the monitor of the analyzer) showing the device's real‐time resonance tracked during the test. b) Plot of the normalized resonant frequency of the device observed for a MAP range over 100 mmHg varied through drug administration and eventual euthanasia.

In order to perform real‐time tracking of blood pressure with the device, another test was conducted using the same pig. Following the same bypass model, another 6 mm diameter graft that contained two fully expanded functional devices (one with 30 mm length and the other with 20 mm length) was studied to compare the dimensional effect on in vivo wireless sensing. A medical pressure transducer (DPT‐248, DELTRANII, Utah Medical Products Inc., USA) was used as a reference sensor to measure the local blood pressure. For this, a 20 gauge tubular catheter was inserted into the artery upstream of the bypass (Figure S7a, Supporting Information), and the other end of the catheter was connected to a transducer linked to the multiparameter physiologic monitor system. This system showed the local arterial blood pressure (read from the reference transducer), its temporal plots, and other vital signs on the monitor. The readout (Figure S7b, Supporting Information) indicates a certain level of variation in the blood pressure read using a hemodynamometer applied to a model's limb (89/42 mmHg) from the local arterial pressure near the graft read with the reference transducer (83/56 mmHg) (it is also worth noting that according to the Bernoulli's principle, the actual blood pressure exerted on the stent device was expected to be larger than the reference transducer as the luminal diameter of the graft was larger than that of the anastomosed carotid artery). The base resonant frequency of the 30 mm stent was wirelessly detected to be 27.96 MHz at a MAP of 59 mmHg (which was 42 MHz in air and 29.5 MHz in saline for this device) using an external antenna. Its reflection coefficient magnitude was higher while frequency was lower, compared to those of the two (functional and control) 30 mm devices expanded in the 5 mm diameter graft from the first test and the 20 mm device expanded in the same 6 mm diameter graft. These results are presumed to be due to a higher inductance of the current device with larger dimensions. This outcome is consistent with the result discussed earlier with Figure 4c. To increase the systemic blood pressure of the swine and wirelessly interrogate the change using the stent device, dobutamine, a vasoconstrictor drug, was administrated to the model. As the drug was delivered, the swine's blood pressure increased and peaked at 138/94 mmHg (a MAP of 108 mmHg) as read by the reference transducer with a maximum pulse rate of 236 per minute. The stent device in the graft responded to this pressure increase with a consistent decrease of resonant frequency by ≈3.5% from its base value of 27.96 MHz (at 59 mmHg MAP), as shown in **Figures** **6**a,b. Finally, the pig was euthanized, and the blood pressure gradually decreased toward 0 mmHg; the device responded to this pressure drop as well, with corresponding rise of its resonant frequency as also shown in Figure 6b. This test verified the effectiveness of the developed smart stent, for real‐time and continuous tracking of local blood pressure change that well covers the range typically seen in human (a MAP of 93 mmHg, with typical blood (systolic/diastolic) pressure of 120/80 mmHg).

## 3 Conclusion

In summary, we have demonstrated advanced smart stent technology using a MEMS‐sensor‐integrated antenna stent with enhanced electromechanical performances. The custom‐designed implantable capacitive pressure sensor chips based on medical‐grade stainless steel were laser‐microwelded on 20 and 30 mm length inductive antenna stents made of the same alloy to form passive resonant *LC* tanks that served as stent‐based wireless pressure sensors. The gold and Parylene C coatings applied to the devices ensured both biocompatibility and X‐ray opacity of them. Moreover, the adopted microwelding integration method provided clear merits in increasing both mechanical robustness and electrical performance of the device. The microfabricated prototypes were robust enough to achieve compatibility with the standard catheter assembly and PCI procedures. The combination of thick gold coating and microwelding integration was highly effective to raise the device's Q factor, which in turn led to significant resolution enhancement in wireless pressure sensing. The prototypes deployed in the vascular grafts were tested to demonstrate wireless monitoring of in‐stent blood pressure using a swine model. These results from both the bench tests and the animal study advance smart stent technology further, toward its clinical evaluation and application for long‐term monitoring of implanted stents. The technologies of robust electromechanical integration and biocompatible packaging developed for the current device can benefit the development of other types of smart medical implants.

## 4 Experimental Section

*Fabrication of Inductive Antenna Stent*: Similar to commercial stent production, the antenna stents were laser‐micromachined from medical‐grade 316L stainless‐steel tubing (with an inner diameter of 1.8 mm and a wall thickness of 100 µm) after which electropolishing was performed to smoothen the machined surfaces. Two tab‐like structures (0.6 × 2.0 mm^2^) were fabricated at both ends of the stent to be used as the platforms for sensor integration. The tubing has to be annealed (either before or after laser patterning) so that the stent can be balloon expandable. Postannealing stainless‐steel of fabricated stents exhibited a yield strength of ≈310 MPa and an elongation of >45%, suitable levels that enabled mechanical functionality of the stent.^32^ After thorough cleaning in sonicated ethanol, the stents were first pretreated through a strike process to deposit thin gold film that serves as an adhesion layer on the stainless‐steel stents, and then subjected to electroplating of 24K gold, using commercially available strike/plating solutions (TriVal‐24K Acid Gold Strike and 24K Bright Gold Plating Solution, respectively, Gold Plating Services, USA). Gold electroplating was performed for a 15–20 µm thickness (with a current of 10 mA for 1 h) to fully exploit the skin effect (considering that the skin depth in gold is up to ≈14 µm for the resonant frequency spectrum (30–100 MHz) adopted for the developed device). The DC resistance of the antenna stents was measured to decrease by 15× (from 22.5 Ω to 1.5 Ω on average) after gold electroplating. Moreover, gold‐covered stents were not only biocompatible and anticorrosive but also had high X‐ray opacity. The fabricated 20 and 30 mm stents (with helical turns of 15 and 23, respectively) were measured to have inductances of 180 and 268 nH (at 10 MHz), respectively.

*Fabrication of Implantable Micropressure Sensor*: The sensor microchip used to prototype the smart stent was constructed on the stainless‐steel substrate with a reference cavity, which was hermetically sealed at atmospheric pressure by bonding a polyimide–metal multilayered diaphragm on the substrate to form a parallel‐plate capacitive pressure sensor. The fabrication followed three main steps, i.e., stainless‐steel chip micromachining, thin sensing diaphragm formation, and thermal bonding and releasing. First, a micromachining technique called micro‐electrodischarge machining (µEDM; EM203, Smaltec International LLC, IL, USA), capable of high‐precision shaping of bulk metals/alloys, was used to 3D pattern a 15 µm deep reference cavity (containing eight dead‐end holes for enhanced sensitivity) for each chip on 200 µm thick foil of 316L stainless steel and then dice each chip from the foil. Next, the diaphragm component was prepared by electron‐beam evaporation of multiple metal layers (100 nm chromium/150 nm gold/50 nm titanium; chromium and titanium used as adhesion layers) on copper foil serving as their temporary substrate, followed by spin coating and curing of 5 µm thick polyimide film on the metal (titanium) surface. The stainless‐steel chips were then thermally bonded on the polyimide side of the diaphragm at 260 °C for 35 min while applying a static pressure (≈60 MPa). After bonding, the excess regions of the polyimide and titanium‐gold layers were removed by oxygen plasma and wet etching, respectively. The fabrication was finalized by releasing the sensor chips by etching away the entire copper foil and chromium layer. The fabricated sensors showed an average sensitivity of 156 ppm mmHg^−1^.

*Laser Microwelding Integration and Device Packaging*: The laser microwelding process was conducted using a commercial system based on a Nd:YAG fiber laser with 1070 nm wavelength (FiberStar Workstation 7600, LaserStar Technologies Co., USA). For this system, a focused laser beam with spot diameters down to ≈25 µm enabled microscale and direct (no filler) welding at the interface between the sensor die and the stent. To minimize the heat‐affected zone and avoid thermal shock to the sensor, this process used the single‐pulse mode (with a peak power and a pulse duration of 130 W and 0.5 ms, respectively) instead of the multiple‐pulse or seam mode. A three‐axis micromanipulator (SM 3.25, Marzhauser Wetzlar GmbH & Co. KG, Germany) was used to precisely position the sensor microchip with respect to the stent's tab platform while they made direct contact with each other. With the above setting, fabricated sensor chips were microwelded onto the stents (passivated by 20 µm thick layer of Parylene C coated on them, to make their surfaces electrically isolated and biocompatible; this film on the tab platform was manually removed prior to welding) by shooting the laser at multiple spots around their interface (Figure 2b,c) with the guidance of an aligning infrared spot. The *LC* circuit was completed by electrically bridging between the sensor's diaphragm and the other side of the stent's tab using a thin (80 µm thick) copper wire. Finally, another 2 µm thick Parylene C film was deposited over the entire device to ensure both electrical insulation and biocompatibility on all exposed surfaces of the device.

*Surgical Model with Vascular Graft*: For this model, a surgical cut‐down to expose the carotid artery was performed on an anesthetized pig (Figure S6b,c, Supporting Information). The carotid artery was dissected out and isolated. Heparin with a dose of 50 IU kg^−1^ was administered intravenously. The mid‐section of the carotid artery was ligated with a 2‐0 silk ligature. Blood flow through the carotid artery was temporarily suspended by vascular clamps placed on the artery. Two separate 12 mm long incisions (arteriotomies) were made in the artery proximal and distal to the silk ligature. The 5/6 mm GORE‐TEX graft with the expanded devices was beveled to fit the length of the arteriotomy and anastomosed in sequence by suturing it with 6‐0 polypropylene vascular sutures to the proximal followed by the distal arteriotomies. Prior to completing the anastomoses, flow was temporarily established to flush out any air or blood clots that may have accumulated in the graft and around the vascular clamps. After completing the anastomoses, the clamps were removed to allow blood to flow through the bypass graft.

## Conflict of Interest

The authors declare no conflict of interest.

## Supporting information

Supplementary
