# Supplementary material for: Enabling Angioplasty‐Ready “Smart” Stents to Detect In‐Stent Restenosis and Occlusion
Source: Adv Sci (Weinh). 2018 Feb 16;5(5):1700560. doi: 10.1002/advs.201700560 (PMC5979749; doi:10.1002/advs.201700560)
Supplement: Supplementary file 1 — Supplementary [file ADVS-5-1700560-s001.pdf]

Copyright WILEY-VCH Verlag GmbH & Co. KGaA, 69469 Weinheim, Germany, 2017.

ADVANCED SCIENCE
Open Access

Supporting Information

for *Adv. Sci.,* DOI: 10.1002/advs.201700560

Enabling Angioplasty-Ready “Smart” Stents to Detect In-Stent Restenosis and Occlusion

*Xing Chen, Babak Assadsangabi, York Hsiang, and Kenichi Takahata**

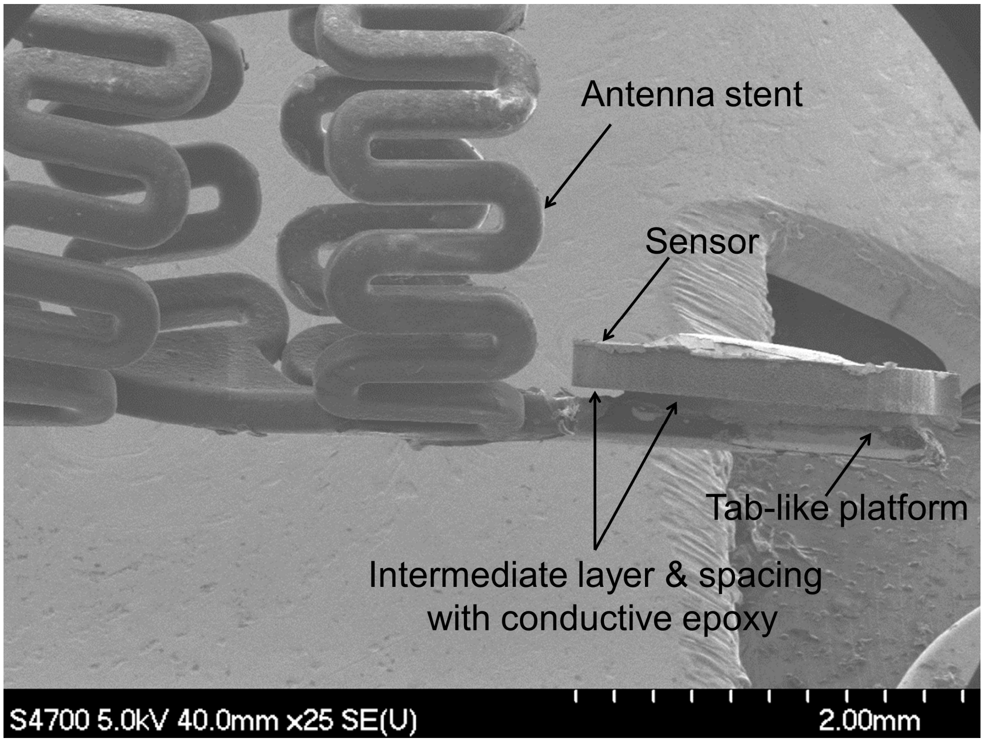

Figure S1. Scanning electron microscope image of an integrated stent device. An example of sensor integration on the tab-like platform of an inductive stent using conductive epoxy adhesive, showing a thick adhesive layer with partly unfilled spacing between them.

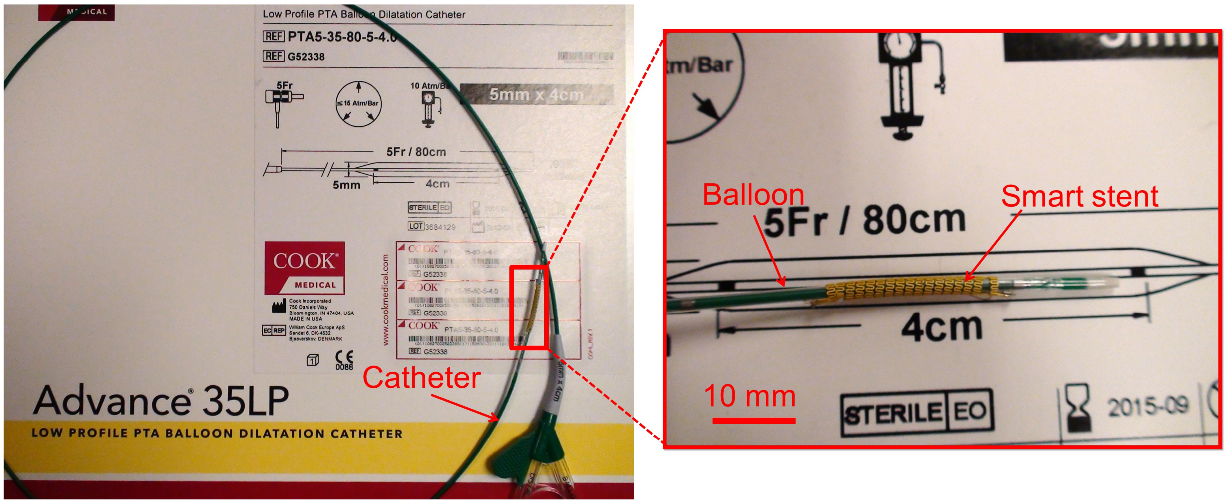

Figure S2. Smart stent assembled on balloon catheter system. A sample of a 20-mm-long smart stent is mounted on a commercial dilatation catheter (Cook Medical Inc., IN, USA) with its 40-mm-long balloon region.

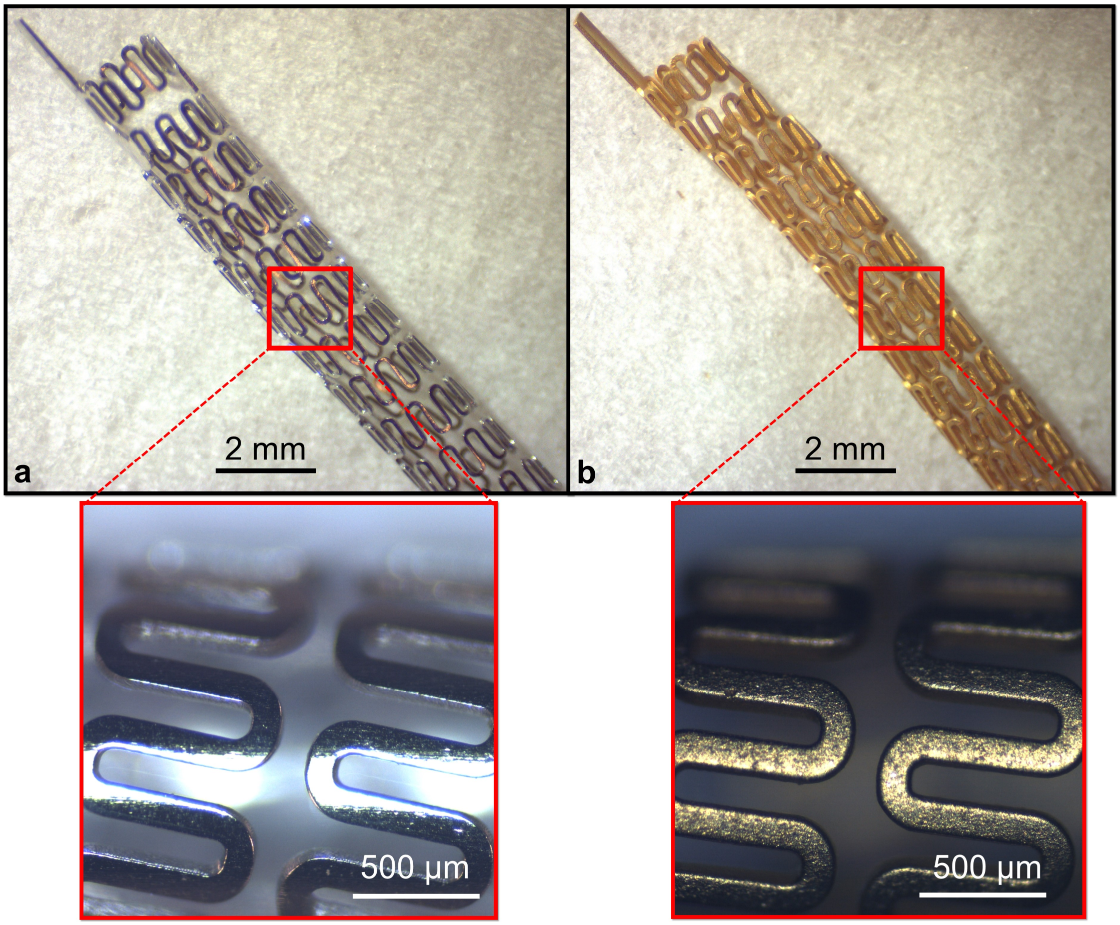

Figure S3. Optical images of antenna stents and their close-ups. a) Bare stainless-steel device. b) Gold-electroplated device providing higher antenna performance with a higher Q factor due to the skin effect.

a

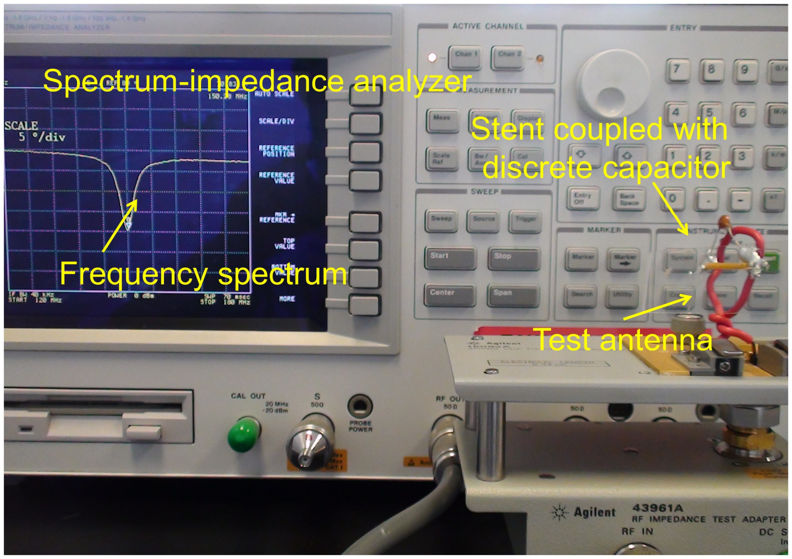

b

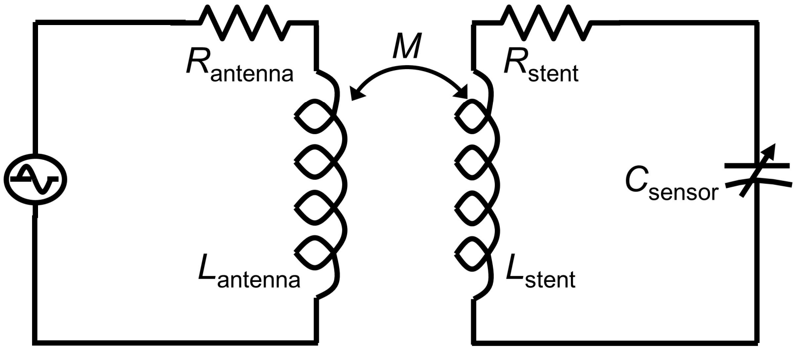

Figure S4. a) Experimental set-up for frequency characterization of inductive antenna stent. The resonant frequency of a sample gold-coated stent resonator (formed with a 10-pF capacitor) is wirelessly measured through inductive coupling with a test loop antenna connected to the analyzer. b) An equivalent electrical setting for the wireless interrogation of the *LC*-tank stent device (right circuit) with the antenna (left circuit) inductively coupled via the mutual inductance, *M*.

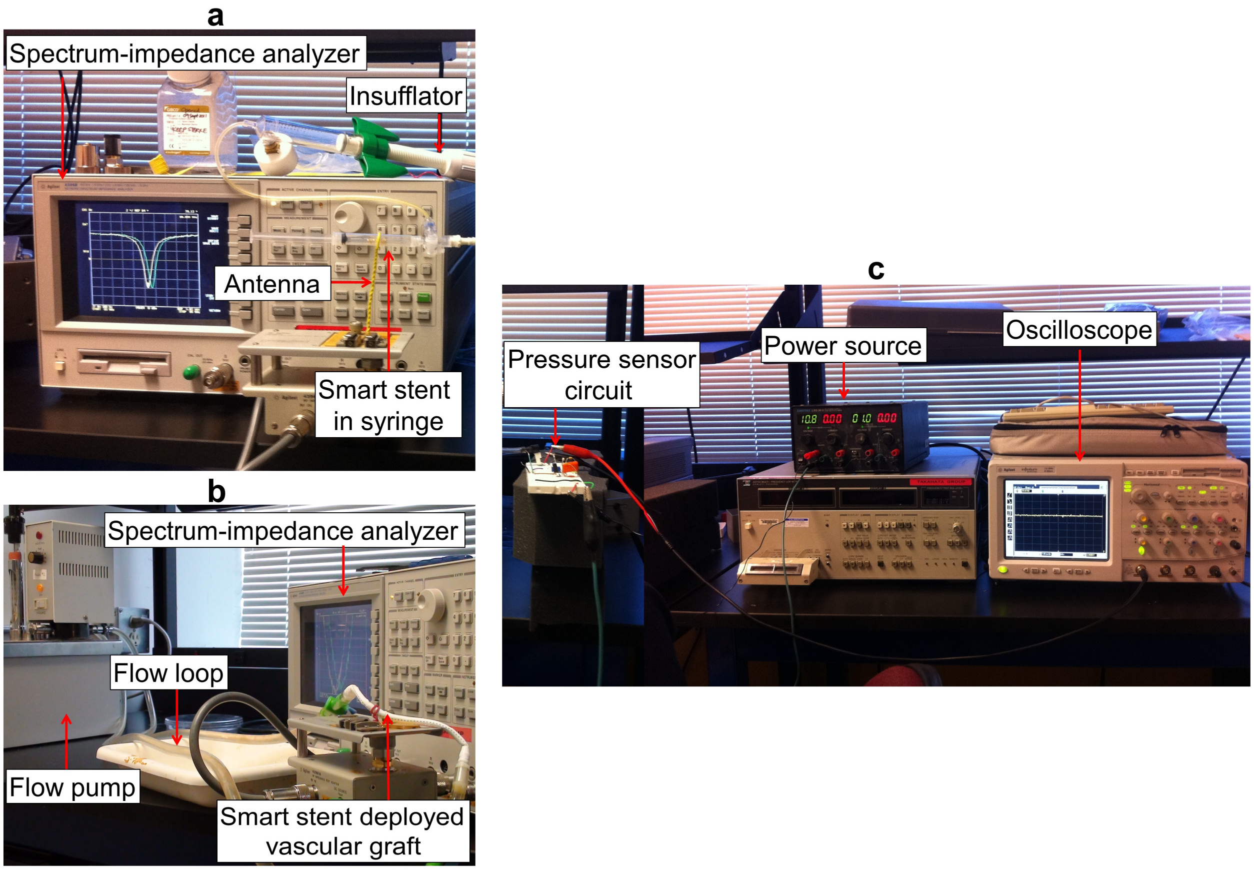

Figure S5. Experimental set-up for wireless characterization of smart stent's response to hydrostatic and hydrodynamic pressures. a) The spectrum-impedance analyzer with loop antenna is used to wirelessly interrogate frequency response of smart stent in an enclosed syringe that is connected to an insufflator and a commercial pressure sensor through a three-way Luer connector. The insufflator is used to change hydrostatic pressure (up to 248 mmHg) of either air or saline in the syringe. b) Flow circulation system to test smart stent-deployed vascular graft in fluid dynamic environment. c) The commercial pressure sensor (24PC, Honeywell Sensing and Control, MN, USA) provides the reference pressure from either syringe or flow loop in a form of voltage signal measured using an oscilloscope (54846B, HP Agilent, CA, USA).

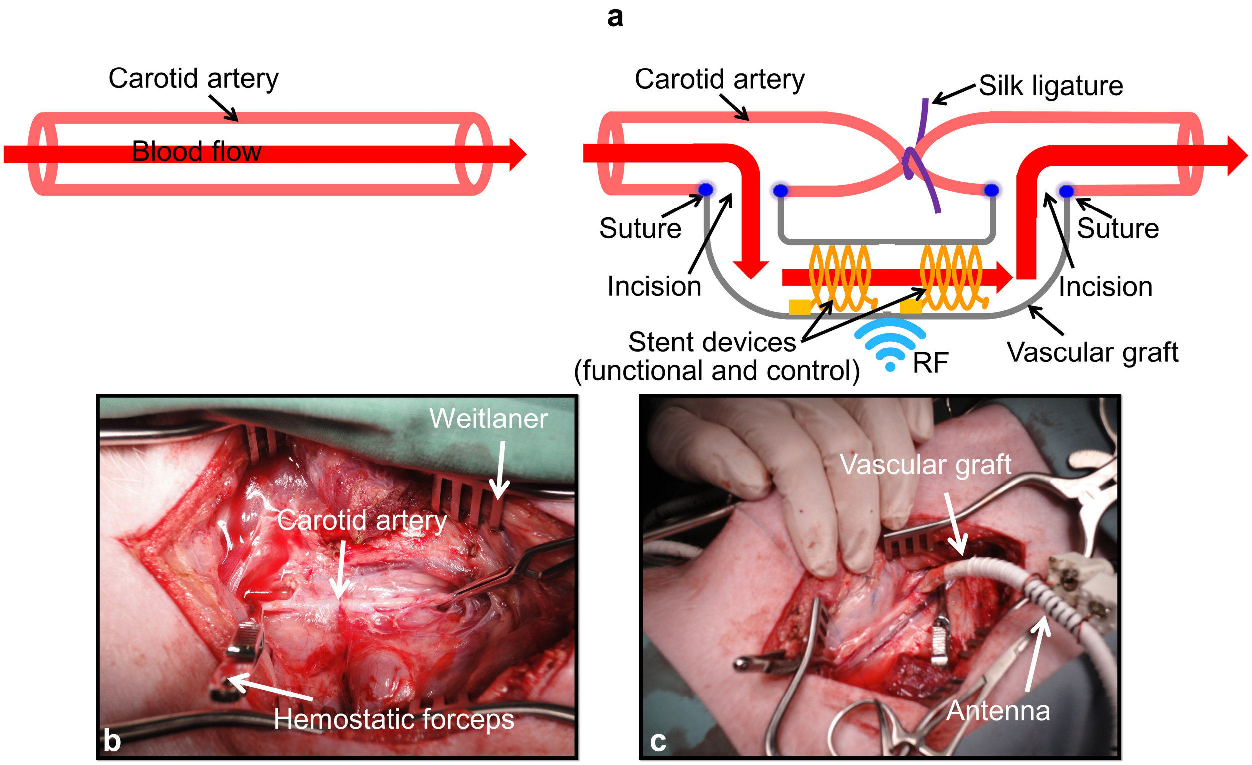

Figure S6. Surgery procedure for bypass model. a) Schematics of the carotid artery before (left) and after (right) graft bypass surgery. The test used a carotid artery that had ~2 mm outer diameter with normal blood flow. After temporarily stopping blood flow through the exposed artery with vascular clamps and tying up the artery using a silk ligature, two incisions were made proximal and distal to the ligature, at which the ends of the vascular graft that contained stent devices were sutured. The clamps were then removed to detour blood flow through the graft as a bypass. b) Exposure of left carotid artery. c) Suturing of one end of device-deployed graft at incision on carotid artery.

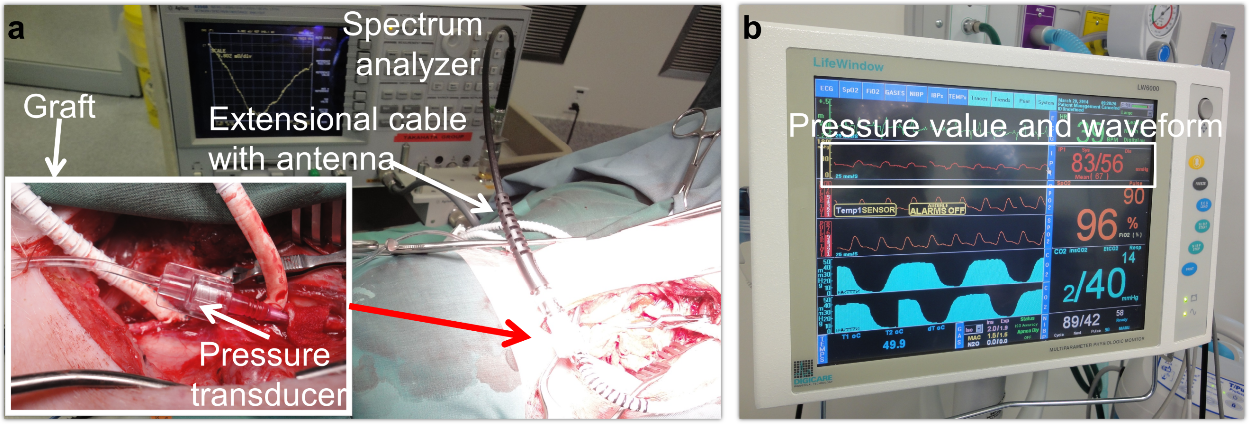

Figure S7. Measurement set-up for animal testing. a) Antenna (connected to spectrum analyzer via extension flexible cable) used to wirelessly read the resonant frequency of sensor-integrated stent deployed inside the graft, and pressure transducer used for referencing local blood pressure value in graft. b) Multi-parameter physiologic monitor providing vital signs of the swine model.

Long-term reliability of stent-sensor interface created using laser microwelding and conductive epoxy.

In our accelerated aging tests in 1× phosphate buffered saline at a temperature of 67 °C (8× compared with the case of normal body temperature at ~37 °C), the laser-microwelded sample (316L stainless steel) showed no failure over a consecutive period of three months, while epoxy jointed sample (same steel) exhibited cracks after 42 days, verifying significant merit of laser microwelding in achieving robust, safe packaging and long-term reliability for smart stent technology.

Wireless sensing principle.

The wireless RF sensing system consists of two main parts, the passive *LC* tank (smart stent, the focus of this study) and the readout unit (commercial spectrum-impedance analyzer in this study). The resonance of a *LC* tank occurs when the capacitive (pressure sensor) and inductive (stent) reactances become equal to each other. We use two frequency-domain telemetry methods to interrogate the tank's resonant frequency that reflects the amount of pressure locally applied to the capacitive sensor integrated on the stent. One method probes the impedance phase of a loop

antenna placed over the inductive stent through inductive coupling made between them. As the analyzer sweeps frequency in the antenna's impedance phase, a dip appears in the spectrum, with an approximate phase amount of $\tan^{-1}(k^2Q)$ (where k is the coupling coefficient between the loop antenna and the inductive stent, and Q is the Q factor of the stent device), when the sweeping frequency matches the resonant frequency of the tank (given the principle, this is often called "phase-dip" method). Thus, a pressure change applied to the on-stent sensor leads to a shift in the phase-dip frequency, which can be used to back-calculate the applied pressure change with a known sensitivity of the sensor. A high Q factor of the stent device as well as a high coupling coefficient are two important factors that enhance the dip signal over a baseline noise and thus improve the sensing distance and resolution. The other method is based on the use of reflection coefficient, the intensity ratio of the reflected electromagnetic wave to the incident one. This method is useful for extending the working range between the external antenna and a readout unit with a coaxial extension cable, which can be essential for *in vivo* tests and real use in clinics.

The wireless sensing ranges with the above two sensing methods are up to ~3 cm and ~1 cm when stent devices are present in air and blood, respectively, using the set-up based on commercial spectrum-impedance analyzer as described in the current paper. The smaller distance in blood is mainly attributed to increased RF damping caused by the conductive liquid ambient. The wireless distance requirement varies depending on the application (e.g., from ~2.5 cm for carotid artery to ~5 cm for coronary artery). There are different routes to extending the distance. One path is to improve the Q factor of the device further. Another path is to use a different telemetry scheme. The time-domain transient resonance (often called "ring-down") method is a promising alternative. This method transmits RF bursts with varying frequencies to a *LC*-tank sensor and analyzes the reflected transient wave to determine the resonant frequency of the tank.[S1] Our preliminary study showed 2-3× distance enhancement using smart stents,[S2] and further development is ongoing.

References

[S1] J. Joy, J. Kroh, M. Ellis, M. Allen, W. Pyle, Santulli, US Patent No. 7245117, 2007.

[S2] D. S. Brox, X. Chen, S. Mirabbasi, K. Takahata, *IEEE Antenn. Wireless Propag. Lett.* 2016, **15,** 754.
